# Community-Based Participatory Research and Drug Utilization Research to Improve Childhood Diarrhea Case Management in Ujjain, India: A Cross-Sectional Survey

**DOI:** 10.3390/ijerph16091646

**Published:** 2019-05-11

**Authors:** Aditya Mathur, Devendra Baghel, Jitendra Jaat, Vishal Diwan, Ashish Pathak

**Affiliations:** 1 Department of Pediatrics, R. D. Gardi Medical College, Ujjain 456006, India; dr.adityamathur121@gmail.com (A.M.); dbaghel201@gmail.com (D.B.); jitendrajat46@gmail.com (J.J.); 2 Global Health—Health Systems and Policy, Department of Public Health Sciences, Karolinska Institutet, SE-171 76 Stockholm, Sweden; vishaldiwan@hotmail.com; 3 Department of Public Health & Environment, R. D. Gardi Medical College, Ujjain 456006, India; 4 Department of Women and Children’s Health, International Maternal and Child Health Unit, Uppsala University, SE-751 85 Uppsala, Sweden

**Keywords:** Child, diarrhea, water sanitation and hygiene, rehydration solution, zinc, case management, antibacterial agents, drug utilization, community participation, India

## Abstract

Childhood diarrhea continues to be a major cause of under-five (U-5) mortality globally and in India. In this study, 1571 U-5 children residing in nine rural villages and four urban slums in Ujjain, India were included with the objective to use community participation and drug utilization research to improve diarrheal case management. The mean age was 2.08 years, with 297 (19%), children living in high diarrheal index households. Most mothers (70%) considered stale food, teething (62%), and hot weather (55%) as causes of diarrhea. Water, sanitation, and hygiene (WASH)-related characteristics revealed that most (93%) households had toilets, but only 23% of the children used them. The study identified ineffective household water treatment by filtration through cloth by most (93%) households and dumping of household waste on the streets (89%). The results revealed low community awareness of correct causes of diarrhea (poor hand hygiene, 21%; littering around the household, 15%) and of correct diarrhea treatment (oral rehydration solution (ORS) and zinc use, 29% and 11%, respectively) and a high antibiotic prescription rate by healthcare providers (83%). Based on the results of the present study, context-specific house-to-house interventions will be designed and implemented.

## 1. Introduction

Globally, diarrhea continues to be one of the major causes of mortality among children aged less than five years [1,2]. India as a country, has the highest number of childhood deaths due to diarrhea with 400,000 deaths annually [2]. Three states of India, including Madhya Pradesh (MP), account for nearly half of India’s childhood diarrhea burden [3,4]. The majority of childhood diarrhea deaths (80%) occur in rural areas, where informal healthcare providers (IHCPs) are the predominant providers [5]. Oral rehydration solution (ORS) and zinc use can prevent 69% of the mortality associated with diarrhea among children aged less than five years [2,5]. Despite this, treatment according to the relevant guidelines (i.e., ORS and zinc use) is provided only to 39% of children with diarrhea, but antibiotics are prescribed to 72% of these children [6]. Studies have suggested that improved case management including increasing the prescription of ORS and zinc and reducing that of antibiotics, addressing social determinants of health and research to identify cost-effective interventions, and promoting equitable access to interventions, is needed [7,8]. Some studies have examined healthcare workforces’ knowledge, attitude, and practice toward diarrhea [9,10,11]. Studies performed in India have shown that parents’ awareness of ORS is high (89%, 86.7%, and 90.7%), but the practice of using ORS for diarrhea episodes is lower (51%, 54.8%, and 60%) in comparison to awareness among parents; these results indicate a wide “know-do” gap [9,10,11].

The aims of this paper are to present data on community perceptions for causes of diarrhea, perceptions for diarrhea treatment, treatments given at home, health-seeking behavior for diarrhea, and the pattern of drug prescription by healthcare providers for diarrhea in the community. Based on this study, interventions would be planned to increase the ORS and zinc prescription rates in the community. 

## 2. Materials and Methods

### 2.1. Study Design and Setting

This cross-sectional community-based study was conducted between June to August 2017. This survey was conducted in the Ujjain district. The Ujjain district is one of the 51 administrative districts in MP. The Ujjain district has a population of 1.9 million within an area of 6091 sq.km; 61% of the district is rural [12]. A list of villages in the Ujjain district and slums in Ujjain city (the district headquarters) was made, and nine villages and four slums were randomly selected for the study.

### 2.2. Sample Size Calculation

For the sample size calculation, the ORS prescription rate was considered the primary outcome. It was assumed that each intervention would increase the ORS prescription rate by 15% at least. National Family Health Survey-4 (NFHS-4) data showed that the ORS prescription rate was 57% in the Ujjain district [13]. Thus, assuming 0.57 as the proportion of healthcare providers prescribing ORS and assuming a 95% confidence interval for this proportion with a width not higher than 15%, the minimum sample size needed was calculated to be 172 children with diarrhea. The study was planned in nine villages and four slums, a conservative estimate of design effect of four was considered appropriate [14]; this gave a minimum sample size needed of 688 children (172 × 4).

### 2.3. Sampling Frame and Data Collection Tools and Methods

All households in each of the selected nine villages and four slums were manually listed to obtain a sampling frame of households. A total of 2830 households were surveyed to list households having children up to five years of age. A total of 1181 households were identified to have children up to five years of age. These households were further surveyed, and 1571 children were included in the study after obtaining informed consent from the family head.

All households in the sampling frame were visited. Mothers or caregivers (maternal or paternal grandmother/grandfather/aunt/uncle) were interviewed by trained data collectors and a predesigned questionnaire was filled-in to obtain information on their perceptions of the causes of diarrhea. Other questions explored their perceptions of treatment; number of diarrhea episodes experienced by their child in the last three months and treatment given for the last diarrhea episode at home. Questions assessed their awareness of ORS and zinc use; availability of ORS and zinc; feeding practices during diarrhea, and details on health-seeking behavior for the last diarrhea episode. The questionnaire collected information on household factors including education status of the mother, caste, religion, type of house, number of household members, and socioeconomic status. Information on water, sanitation, and hygiene (WASH)-related variables were also collected from each household.

The questionnaire was first prepared in English, then translated to Hindi, and back-translated by subject and Hindi language experts [15]. Any discrepancy was resolved by reaching a consensus. For assessing content and construct validity [15], the questionnaire was pilot tested on 50 respondents. Changes were made to three questions related to household WASH characteristics and health-seeking behavior for diarrhea, which had an intra-class correlation coefficient of less than 0.65.

The proportion of children prescribed ORS and zinc, the timing of ORS and zinc initiation post diarrhea, and duration of ORS and zinc use was calculated through house-to-house surveys. The antibiotic prescribed for each diarrhea episode for a given child was also noted. To get an accurate account of antibiotic use in the previous episode of diarrhea, mothers/caregivers were asked to show the original prescriptions, package inserts and partly used packages of all drugs consumed. Further, the mothers were shown a picture of 20 commonly marketed antibiotic syrups. Injectable antibiotic use was ascertained by interviewing the formal and informal health care providers catering to the study population. If the mothers/caregivers could not identify the specific antibiotic using the above three methods, then such an incident was classified as “non-use” of antibiotic. 

The diarrhea index was calculated by summing the number of diarrhea episodes in the previous three months among all children aged less than five years residing in a given household divided by the total number of children aged less than five years and multiplied by 100, as shown in the formula (1) below:-

(1) $$\mathrm{Diarrhea}\;\mathrm{index}=\frac{\sum a_{n}+b_{n}+c_{n}+d_{n}}{y}\times 100$$

where,

*1.*  *a_n_* = number of episodes of diarrhea in the past three months in the first child living in the same household.
*2.*  *b_n_* = number of episodes of diarrhea in past three months in the second child living in the same household as a.
*3.*  *c_n_* = number of episodes of diarrhea in the past three months in the third child living in the same household as a and b.
*4.*  *d_n_* = Number of episodes of diarrhea in the past three months in the fourth child living in the same household as a, b, and c
*5.*  *y* = Number of children aged less than five years living in the same household

Households with a diarrhea index of 200 or more were considered to be households with a high diarrhea index (HDI). The cut-off for HDI was decided post-hoc based-on the results of the study. All HDI households are the targets of community-based interventions to improve diarrhea case management in the future.

### 2.4. Data Management and Data Analysis

The data was collected on the paper-based questionnaire. A database was created using Epidata 3.1 (The EpiData Association, Odense, Denmark). Data entry was performed by trained research assistants and was supervised by senior researchers. All data entered were double-checked for quality assurance. All continuous variables that follow a normal Gaussian distribution were presented as mean ± standard deviation. All categorical variables were summarized and expressed as proportions. Data analyses were performed using Stata (Version 13.0, Statacorp., College Station, TX, USA).

### 2.5. Ethical Consideration

This study was approved by the Institutional Ethics Committee (IEC) of the R.D. Gardi Medical College, Ujjain, India (Approval number: IEC/RDGMC/493). Informed consent was obtained from participants before the interview. Participants had the right to withdraw from the study at any time.

### 2.6. Availability of Data and Materials

Due to ethical and legal restrictions, all inquiries should be made to The Chairman, Ethics Committee, R.D. Gardi Medical College, Agar Road, Ujjain, India 456006 (E-mails: iecrdgmc@yahoo.in, uctharc@sancharnet.in), giving full details of the intended use. Upon verification of genuineness of the inquiry, the data that will be generated from various studies of this protocol will be made available. For reference, please quote the ethical permission number: 2016/01/18-493.

## 3. Results

**Figure 1:**
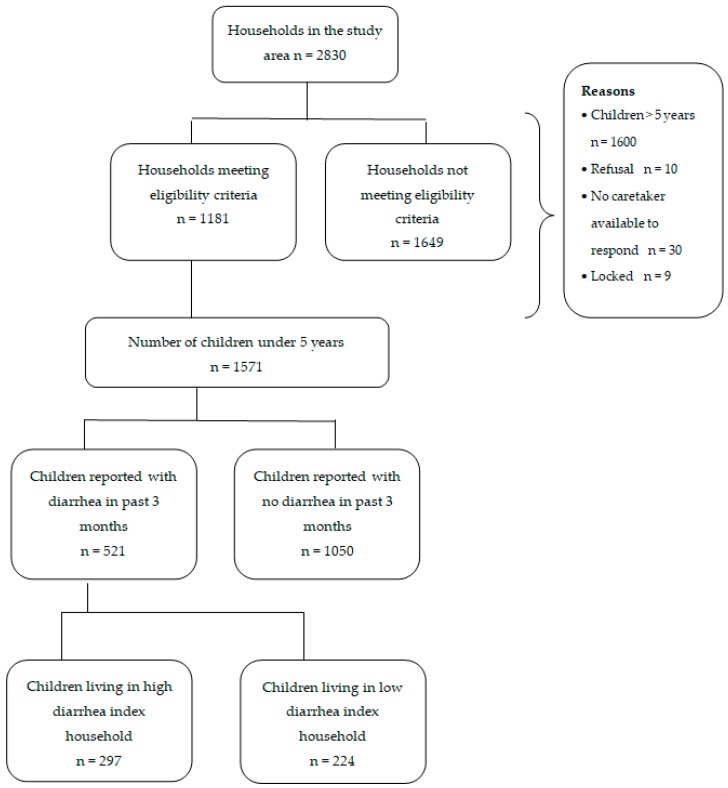
Process of selection of the study participants and their distribution according to low and high diarrheal index households in the study.

A total of 2830 households were approached and 1181 households, which had children below five years of age, were included in the study. From the 1181 households, 1571 children were included, with 815 boys and 756 girls. A total of 858 children belonged to rural areas, and 713 were from urban slums. The selection of study participants and their distribution according to low and high diarrheal index households is depicted in Figure 1. In the study area, 17 formal and 43 informal healthcare providers are present, which were often the first point of contact for healthcare-seeking for the community. Mothers were the main caregivers in the majority (99.6%) of households.

Table 1 provides the socio-demographic characteristics of the study participants.

**Figure 2:**
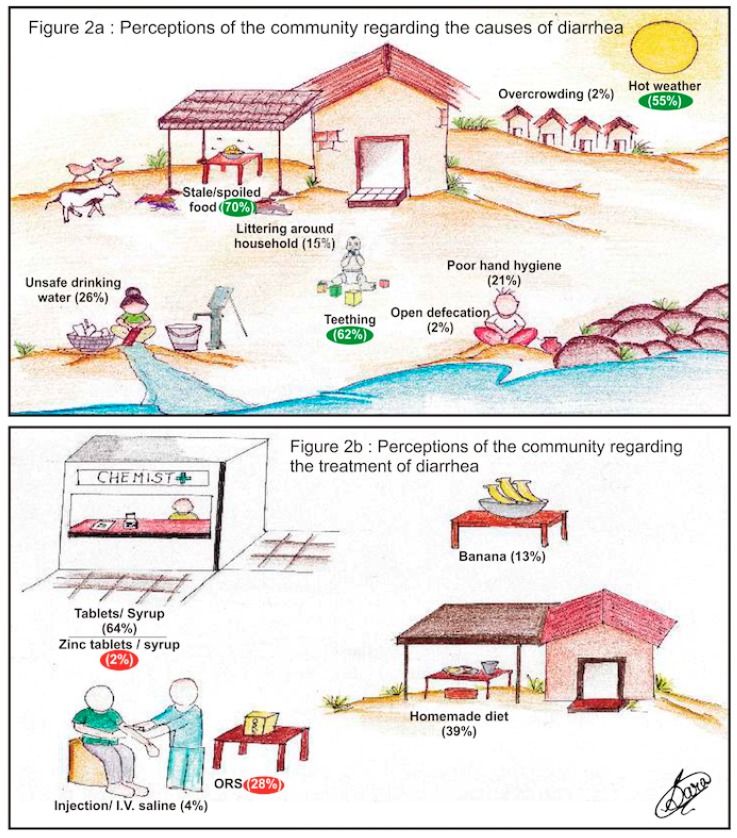
Perceptions of 1571 mothers regarding cause (**a**) and treatment of diarrhea (**b**) (Table S1 provides details in tabular form).

Table 2 provides the WASH characteristics of the households included in the study. Figure 2a depicts the community perceptions of the causes of diarrhea and Figure 2b depicts the community perceptions of various effective treatments (Supplementary Table S1). 

### Treatment Given to Children for the Last Episode of Diarrhea

**Figure 3:**
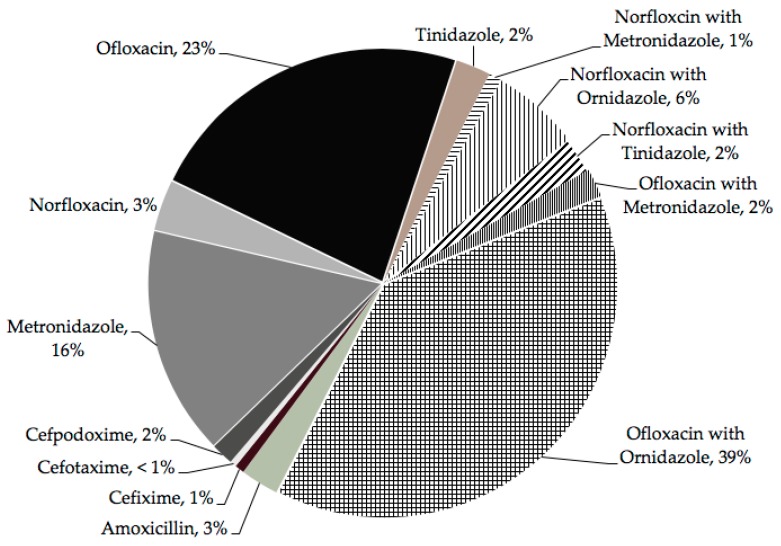
Antibiotics prescribed to the children with diarrhea during the last episode of diarrhea.

Out of the total 1571 children, 521(33%) children were reported to have acute diarrhea. Among the 521 children having diarrhea, a total of 510 (98%) children received some treatment (Table 3). The different antibiotics prescribed to children during the last episode of diarrhea are shown in Figure 3. The most common antibiotic prescribed was ofloxacin (*n* = 104; 23%), followed by metronidazole (*n* = 72; 16%), and the most commonly prescribed combination of antibiotics was ofloxacin with ornidazole (*n* = 175; 39%), followed by norfloxacin with ornidazole (*n* = 28; 6%). Overall antibiotic combinations were prescribed to 54% of children (*n* = 227). Among the children who received treatment (*n* = 423), 83% received antibiotics, 29% received ORS, and only 10% received zinc. Other drugs prescribed were probiotics (25%), paracetamol (18%), loperamide (16%), dicyclomine (7%), and ondansetron (5%). Figure 3 provides the details of the antibiotics given to children with diarrhea. 

## 4. Discussion

The study included a total of 1571 children residing in 1181 households located in both rural and urban areas of Ujjian. The mean age of children included in the study was 2.08 years. The WASH-related characteristics revealed that most (60%) households used hand-pump as a drinking water source, and most (71%) households filtered water before storage using a simple cloth. Many (93%) households had a toilet built in the household, but only 23% of children used them. Most (89%) households dumped their household waste on the streets. Almost all (98%) mothers claimed to wash hands after toilet use, but only 30% did so before cooking. Most (70%) mothers perceived that stale or spoiled food followed by teething (62%) and hot weather (55%) were the most common causes of diarrhea. For the treatment of diarrhea, the perception was that some form of tablets/syrups were prescribed (64%), children given homemade diet (35%), and bananas were perceived to be beneficial by 13% of mothers. Nearly half the mothers continued breast-feeding during the diarrheal episode; however, only 33% and 12% of the mothers had heard about ORS and zinc, respectively. Of the children that received treatment, antibiotics were prescribed in 83% and ORS and zinc in 29% and 11%, respectively.

In the present study, the major cause of diarrhea perceived by the community was stale food, followed by teething and hot weather. Studies conducted in Eastern Sudan [16], Saudi Arabia [17], and Nepal [18] have also reported stale food and teething as the major risk factors for diarrhea, as identified by the community. In the three aforementioned studies, hygiene and sanitation-related risk factors including, poor hand washing practices, open-air defecation, and littering around the household were perceived as less important causes of childhood diarrhea by the community [16,17,18], which is similar to the present study.

In India, diarrhea seasonality is well known [3,19]. Rotavirus infections present two seasonal peaks in India, one in winter and the other in summer; the increase in temperature reduces the transmission of rotavirus infections [20]. In summer, the limited potable water supply poses potential public health risks for diarrhea [3]. The scarcity of potable water during hot Indian summers and the peaking of diarrhea during summers probably shapes the community’s perception that hot weather is an important risk factor for diarrhea. 

The community perceived unsafe drinking water as an important cause of diarrhea (26%). The majority of households that reported household water treatment used simple filtration of drinking water through a cloth. According to World Health Organization and United Nations International Children's Emergency Fund’s core questions on drinking water and sanitation for household surveys [21], simple filtration is classified as an inadequate method; however, it has been shown to be effective in protecting against *Vibrio cholera* infections in 65 villages in Bangladesh, in which simple “sari” cloth was used for water filtration [22]. Most households in the study area had access to safe drinking water sources. Fecal contamination of drinking water remains the most important cause of diarrhea globally, although this was not specifically tested in the present study [23]. Contamination of drinking water usually occurs not at the source but mostly at the household level [24]. Littering around the household can play a major role, as perceived by the community. The nonuse of toilets, even when available within households, is a problem that can be addressed through behavior change communication (BCC) and has been reported in rural areas of Odisha, India [25] and urban areas of Bhopal in MP [26]. Other resource-poor countries, such as Bangladesh [27], Zambia [28], and Ethiopia [29], also show low utilization rates of latrines.

In this study, the majority (64%) of mothers/caregivers (Figure 2b) perceived that syrups and tablets provided by healthcare providers are effective treatments for diarrhea. The knowledge of ORS and its benefits for childhood diarrhea was poor in our settings (28%, Figure 2b), which is in contrast to the findings of other studies conducted in India [9,10,11]. In our setting, the zinc use rate of 2% was much lower than the national average rate of approximately 30% [3]. Moreover, there was a severe lack of knowledge of zinc use among mothers in India and other resource-poor countries [30,31,32]. This lack of knowledge and low acceptance for ORS and zinc use among community members/mothers are prevalent despite the fact that ORS and zinc tablets are offered free of cost by the government health sector and are offered at the doorstep by front-line healthcare workers in India. However, the increased use of homemade fluids and bananas is encouraging and should be promoted [33]. A high proportion of antibiotic prescriptions, especially the irrational combination of antibiotics, (most commonly ofloxacin with ornidazole) for diarrhea has been documented and is a major public health problem in India [6]. High use of antibiotics for diarrhea in the study settings warrants specific interventions for healthcare workers. 

### Methodological Considerations

In the present study, we attempted to retrospectively ascertain drug use mainly ORS and zinc and antibiotics through interviews, which may be subject to bias due to differential recall. ORS and zinc use recall were better than antibiotic recall as participants could recognize ORS and zinc easily. This kind of recall bias would underestimate antibiotic use more compared to ORS and zinc use.

## 5. Conclusions

Childhood diarrhea is a major public health concern globally and in India. Effective interventions are known but are rarely implemented in resource-limited settings and settings with fragmented healthcare systems, similar to the settings in this study. The results of this study provided insights into community awareness of causes of diarrhea, its treatment, and other WASH-related variables. The results of the present study will help the design of content-specific interventions at the community level for the mothers, children, and healthcare workers.

## Figures and Tables

**Table 1:** Socio-demographic characteristics of 1181 households having 1571 children included in the survey.

Socio-demographic characteristics.	Mean	SD
Continuous variables		
Age of the children (years)	2.08	1.18
Family size (number)	7.20	3.24
Age of mother (years)	25.11	4.67
**Categorical variables**	*n* = 1181 households	%
**Location**		
Rural	660	56
Urban	521	44
**Education status of mother**		
Uneducated	240	20
Primary	629	53
Secondary or more	312	27
**Caste**		
General	151	13
Scheduled castes *	166	14
Scheduled tribes *	815	69
Other backward class *	49	4
**Religion**		
Hindu	695	59
Muslim	460	39
Others	26	2
**Type of home**		
Self-owned	1091	92
Rented	90	8
**Number of household members**		
≤4	254	21
5–8	586	50
≥9	341	29

* For details see pages 10–11 of [12].

**Table 2:** Water sanitation and hygiene-related characteristics of 1181 the households.

Water Sanitation and Hygiene Related Characteristics	*n* = 1181	%
**Water treatment**		
**Drinking water source** *		
Hand-pump	709	60
Bore well	683	58
Tap (municipal supply)	294	25
**Storage practices** *		
Roof-top storage	242	20
Ground storage	1181	100
**Drinking water storage containers** *		
Earthenware pot (*Matka/Ghada*)	1181	100
Buckets	557	47
Plastic cans	298	25
**Frequency of cleaning ground water containers**		
Daily	779	66
Every 2nd–3rd day	250	21
Weekly or more	154	13
**Do you treat water before drinking?**		
No	283	24
Yes	898	76
Filtration using cloth	839	71
Coagulation, flocculation, and sedimentation (by alum)	43	4.4
Boiling for 20 min	16	1.6
**Sanitation**		
**Toilet constructed in household**		
Yes	1100	93
Toilet used by adults	1080	91
Toilet used by children	276	23
**Household waste**		
Thrown on streets	1050	89
Burnt	83	7
Collected and disposed (municipal facility)	48	4
**Hand washing done by mothers/caregivers**		
After cleaning child’s feces	1155	98
After toilet	1153	98
After cooking	960	81
After cleaning child’s urine	675	77
Before feeding child	603	51
To clean visible dirt	558	47
Before cooking	352	30
After cleaning nose/mouth	319	27

* Numbers and percentages totaling more than 1181 and 100%, respectively due to multiple responses.

**Table 3:** Feeding practice and treatment received by 521 (33%) children out of the total 1571 children during the last episode of diarrhea.

Categorical Variables	*n* = 521	%
**Feeding Practices** *		
Continued breastfeeding	249	48
Top milk	270	52
Tea	230	44
Homemade diet	314	60
Mashed food/fruit	224	43
Heard about ORS	173	33
**Where to get ORS?**		
Health care workers	388	74
Pharmacy store	29	6
Don’t know	104	20
Heard about zinc	62	12
**Where to get zinc?**		
Health care workers	177	34
Pharmacy store	29	6
Don’t know	315	60
**Treatment practices**		
No treatment	11	2
Self-treatment only	155	30
Self-treatment with ORS	28	18
Self-treatment with left over medicines	123	79
Zinc tablet/ syrup	6	4
Homemade solutions	34	22
Treatment at healthcare facility	485	93
Government setting	83	17
Private setting	339	70
Formal health care provider	106	31
Informal healthcare provider	233	69
Medical store	63	13
Both self and healthcare facility	130	25
Received any treatment	510	98
Received an antibiotic	423	83
Received ORS	150	29
Received zinc tablets/syrups	54	11

* Numbers and percentages totaling more than 521 and 100%, respectively due to multiple responses.

## Supplementary Materials

The following are available online at https://www.mdpi.com/1660-4601/16/9/1646/s1, Table S1: Community perception about cause and treatment of diarrhea.
