# Supplementary material for: Community-Based Participatory Research and Drug Utilization Research to Improve Childhood Diarrhea Case Management in Ujjain, India: A Cross-Sectional Survey
Source: Int J Environ Res Public Health. 2019 May 11;16(9):1646. doi: 10.3390/ijerph16091646 (PMC6539114; doi:10.3390/ijerph16091646)
Supplement: Supplementary file 1 [file ijerph-16-01646-s001.pdf]

Table S1: Community perception about cause and treatment of diarrhea

**Perception regarding cause of diarrhea**	Number	%
Stale /Spoiled food	830	70
Teething	728	62
Hot weather	648	55
Unsafe drinking water	310	26
Poor hand hygiene	273	21
Littering around household	173	15
Spicy food	43	4
Not vaccinating the child	32	3
Overcrowding	29	2
Open defecation	25	2
**Perception regarding treatment of diarrhea**	Number	%
Tablet/Syrups	751	64
Homemade diet	460	39
O.R.S.	330	28
Banana	158	13
Do Not Know	119	10
Solution of salt sugar	91	8
More water to drink	69	6
Alternative medicines (AYUSH)	53	4
No treatment required	51	4
I.V. / Saline	43	4
Zinc table/syrup	22	2
